# Heart transplantation and human immunodeficiency virus–navigating drug-drug interactions: a case report

**DOI:** 10.1186/s12981-023-00551-x

**Published:** 2023-08-11

**Authors:** Thamer A. Almangour, Preston T. Skersick, Amanda Corbett, Jo E. Rodgers, Patricia P. Chang, Claire E. Farel

**Affiliations:** 1 Department of Clinical Pharmacy, College of Pharmacy, King Saud University, Riyadh, Saudi Arabia; 2 Department of Pharmacotherapy and Experimental Therapeutics, UNC Eshelman School of Pharmacy, Chapel Hill, USA; 3 Department of Medicine, Division of Cardiology, University of North Carolina School of Medicine, Chapel Hill, USA; 4 Department of Medicine, Division of Infectious Diseases, University of North Carolina School of Medicine, Chapel Hill, USA

**Keywords:** Heart transplantation, Human immunodeficiency virus, Case report

## Abstract

**Background:**

Antiretroviral therapy (ART) has led to a decline in human immunodeficiency virus (HIV)-related mortality, but comorbidities, including organ dysfunction, are increasingly the focus of care. Heart transplant (HT) is a very effective therapeutic strategy for end-stage heart failure (HF); however, clinicians may be hesitant due to concerns of complex drug-drug interactions (DDIs) between ART and HT immunosuppressive regimens and the potential impact of ART on long-term HT outcomes. In this report, we describe long-term (76-month) follow-up of a patient with HIV-positive status who underwent orthotopic HT with special emphasis on complex drug interactions.

**Case presentation:**

A 58-year-old man with HIV-1 developed ischemic cardiomyopathy, progressed to end-stage HF and underwent orthotopic HT. To avoid DDIs with planned immunosuppressive therapies, the ART regimen was modified to consist of lamivudine, tenofovir disoproxil fumarate, rilpivirine, and raltegravir. Following HT, the patient’s immunosuppression consisted of tacrolimus and mycophenolate mofetil. He has had normal cardiac function and no opportunistic infections and was subsequently switched to tenofovir alafenamide, emtricitabine, and bictegravir in combination for convenience. Serial HIV-1 RNA blood levels were constantly below the limit of quantification, and his CD4 count remained above 200 cells/mm^3^ (30–35%). Several DDIs were identified and addressed; however, his long-term post-HT complications included one episode of asymptomatic acute cellular rejection, adenocarcinoma of the prostate, basal cell carcinoma, cardiac allograft vasculopathy, and peripheral neuropathy.

**Conclusion:**

The clinical outcome of this case supports the conclusion of previously published reports, summarized here within, demonstrating that HIV-1 positive status should not preclude HT in carefully selected individuals. Both addressing potential DDIs prior to HT and long-term monitoring for routine post-transplant complications and secondary and incidental malignancies are imperative.

## Background

Since the advent of combination antiretroviral therapy (ART) in the mid-1990s, morbidity and mortality associated with human immunodeficiency virus (HIV) have declined [1]. Morbidity and mortality among people with HIV reflect comorbidities common in aging populations, with accelerated rates of vascular disease, including cardiovascular disease (CVD) often attributed to some ART and to HIV itself [2, 3]. CVD has become among the leading causes of non-HIV-related deaths in people with HIV [4]. Common HIV-associated cardiovascular manifestations include hypertension, coronary heart disease, dilated cardiomyopathy, pulmonary hypertension, myocarditis, pericardial effusion, and drug-related cardiotoxicity [5, 6]. Relative to the general population, the prevalence of chronic heart failure (HF) in people with HIV is significantly higher; however, the prevalence of end-stage HF in people with HIV has not been identified [7, 8].

People with HIV were not generally considered transplant candidates due to theoretical concerns for the impact of immunosuppressant medications following transplant, and complex drug-drug interactions (DDIs) between immunosuppressants and ART. In addition, ethical concerns existed regarding the expansion of the selection criteria for organ transplant to include patients for whom outcomes and survival data were uncertain. However, data from kidney and liver transplant recipients have demonstrated that HIV-positive status in selected patients does not adversely affect outcomes [9, 10]. Current evidence from case reports and case series for heart transplantation (HT) in people with HIV has shown similarly promising outcomes (Table 1). Therefore, HT has been performed in select people with HIV with end-stage HF; however, additional data are warranted to evaluate both long-term outcomes. Here we report the long-term follow-up of a person with HIV who underwent orthotopic HT with special focus on DDIs and long-term outcome. The follow-up for this case report is longer than the vast majority of those previously reported and comprises of more contemporary HIV and HT regimens. The patient provided written informed consent for this publication.

**Table 1:** Summary of the previously published cases of people with HIV undergoing HT

Ref #	Year of HT/Country	Gender/Age at HT (years)	Indication for HT	ARV regimen after HT	Routine immunosuppression regimen	Rejection/Grade	Follow-up (months)	Post-HT AIDS-defining illness	Post-HT viral load (copies/ml)/CD4 + count (cells/µl)	Drug interaction/Interacting drugs	Patient Status at last follow-up
[17]^1^	1997/USA	F/>40	Non-ischemic CM	ZDV/3TC/EFV	CYA/MMF/P	Yes/3A and 1 A ^2^	120	No	< 50/>200	NR	Alive
[18]	2001/USA	M/39	daunorubicin associated CM	ZDV/3TC/RTV	CYA/P/MMF or AZA	Yes/2-3 A ^2^	24	No	< 50/multiple episodes of < 200	Yes/CYA and RTV	Alive
[19]	2001/USA	M/42	Idiopathic CM	TDF/3TC/NVP	NR	Yes/2 ^2^	24	No	< 50/650–1000	NR	Alive
[20]^3^	2001–2008/USA	6 M and 1 F/19–48	Dilated CM	NR	CYA/MMF/P	Yes/1B-3 A ^2^	3–88 (mean of 30)	No	UD/>200 (mean of 554 ± 169)	Yes/CYA and HAART	Alive
[21]	2007/Spain	M/39	Ischemic CM	ZDV/3TC/ABC	FK/MMF/MP	Yes/3A ^2^	36	No	< 50/201–754	No	Alive
[22]^1^	2007/USA	M/>60	Ischemic CM	TDF/ FTC/EFV	FK/MMF/P	Yes/moderate	34	No	UD/50 at 30-month	NR	Dead
[23]	2008/France	M/32	Dilated CM	ZDV/3TC/RAL/enfuvirtide	NR	Yes/2R ^4^	30	No	UD/1,560 at 30-month	No	Alive
[24]	2008/USA	M/47	Non-ischemic dilated CM	NR	NR	No	31	No	NR	NR	Alive
[25]	2009/USA	F/42	Non-ischemic dilated CM	NR	FK/MMF/P	Yes/NR	24	No	NR	No	Alive
[26]	2009/Italy	M/36	Non-ischemic dilated CM	ZDV/3TC/EFV	CYA/EVR/MP	Yes/2R ^4^	36	No	UD/>800	Yes/EVR and EFV	Alive
[27]	2011/Italy	M/42	Dilated CM	NR	NR	No	19	NR	NR	NR	Alive
[28]^5^	2000–2016/14 sites USA and Europe	15 M and 6 F/27–61	Non-ischemic CM: 17; ischemic CM: 4	INSTI-based: 8 PI-based: 4 NNRTI-based: 8 Other: 1	FK: 16 CYA: 5 Most received MMF and P	Yes: 14/mild = 6; moderate = 8)	6-123 (median of 35)	1^6^	UD/median of 411 at 1 year	Yes: 4 possible/PI and CNI	Alive (64%)

1. Seroconversion to HIV-positive status occurred after HT

2. The 1990 International Society for Heart and Lung Transplantation (ISHLT) grading for acute cellular rejection on endomyocardial biopsy

3. This is a case series describing 7 HT recipients. Five patients were diagnosed with HIV before HT and 2 patients had seroconversion at 1 and 7 years after HT

4. ISHLT revised grading for acute cellular rejection (ISHLT-2004)

5. This is a case series describing 21 HT recipients

6. Due to cessation of antiretroviral agents (non-adherent patient)

**Abbreviations**

ABC, abacavir; ARV, anti-retroviral; AZA, azathioprine; CM, cardiomyopathy; CNI, calcineurin inhibitors; CYA, cyclosporine; EFV, efavirenz; EVR, everolimus; F, female; FK, tacrolimus; FTC, emtricitabine; HAART, highly active anti-retroviral therapy; HIV, human immunodeficiency virus; HT, heart transplantation; INSTI, integrase inhibitors; M, male; MMF, mycophenolate mofetil; MP, methylprednisolone; NNRTI, non-nucleoside reverse transcriptase inhibitors; NR, not reported; NVP, nevirapine; P, prednisone; PI, protease inhibitors; RAL, raltegravir; RTV, ritonavir; TDF, tenofovir disoproxil fumarate; UD, undetectable; USA, United States; ZDV, zidovudine; 3TC, lamivudine

## Case presentation

A 58-year-old male with HIV-1 infection diagnosed in 1985 with a medical history of hypertension, hyperlipidemia, coronary artery disease, paroxysmal atrial fibrillation and atrial flutter, cleared hepatitis C virus infection, and major depressive disorder presented with severe chronic systolic HF due to ischemic cardiomyopathy initially diagnosed in 2008. His past surgical history included coronary artery bypass graft, biventricular implantable cardiac defibrillator, and abdominal aortic aneurysm repair. He quit smoking in 2011 and denied illicit drug use. He drinks alcohol socially and lives with his male partner. His maternal family history was significant for mitral valve prolapse (eighth decade of life) and paternal history of myocardial infarction and prostate cancer (sixth and seventh decades of life, respectively). He has an allergy (rash) to atazanavir and efavirenz.

Since November 2014, the patient had cardiac functional decline, progressing from New York Heart Association (NYHA) class III to IV with an ejection fraction of 20%. He underwent inpatient evaluation for transplant/left ventricular assist device and initially stabilized well on chronic home infusion of milrinone. With further decline, he was rehospitalized, supported with two inotropes (milrinone 0.375 mcg/kg/min and dobutamine at 1 mcg/kg/min), approved and listed for transplant as United Network for Organ Sharing (UNOS) status 1 A. Notably, he was on long-standing hormone replacement therapy for low serum testosterone, and opted to continue therapy despite potential cardiovascular risks.

After he was diagnosed with HIV in 1985, the patient was initially treated with zidovudine. His subsequent treatment history included primarily nucleoside reverse transcriptase inhibitors (NRTI) and protease inhibitors (PI). He briefly took efavirenz but experienced a rash. His CD4 count was > 400 cells/mm^3^, viral load was largely suppressed with a few interruptions in therapy due to side effects, and he had no history of opportunistic infections. From 2006 to 2015, he took lamivudine, zidovudine, tenofovir disoproxil fumarate, and darunavir/ritonavir. He tried co-formulated tenofovir disoproxil fumarate, elvitegravir, and cobicistat with darunavir in 2013 but felt that it worsened his neuropathy and he resumed his prior regimen. When he was admitted to the hospital for advanced HF in March 2015, his ART regimen was changed to eliminate DDIs with the projected post-HT immunosuppressive regimen. His prior genotypes showed primarily NRTI mutations (D67N, K70R, M184V, and K219Q) without non-nucleoside reverse transcriptase inhibitor (NNRTI) resistance or significant PI-compromising mutations. ART was switched to tenofovir disoproxil fumarate 300 mg po daily, lamivudine 300 mg po daily, rilpivirine 25 mg po daily and raltegravir 400 mg po twice daily in March 2015 for pre-transplant consideration. This regimen was selected primarily to avoid protease inhibitors and maximize tolerability. The patient was actively listed for HT after undetectable viral load was confirmed in May 2015, with a CD4 count of 699 cells/mm^3^ (33%) at that time.

**Fig. 1:**
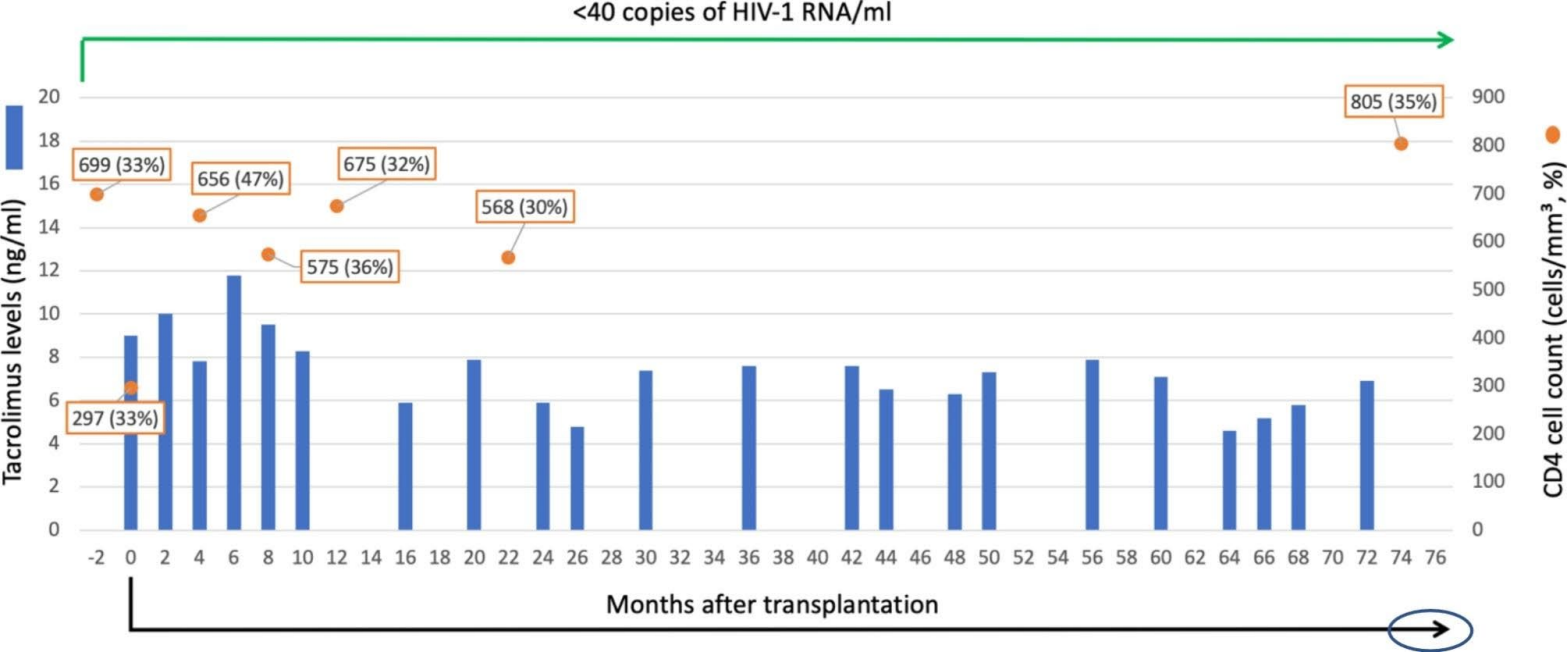
CD4 + T-cell count (%), HIV-1 viral load, and tacrolimus trough levels from heart transplantation to 76 months follow-up

He underwent orthotopic HT in July 2015. Donor was HIV negative. Both donor and recipient were cytomegalovirus (CMV) and Epstein-Barr virus immunoglobulin G (IgG) positive. The recipient was herpes simplex virus 1 and 2 and varicella zoster virus IgG positive. Toxoplasma serology were negative for both donor and recipient. He was started on ganciclovir 2.5 mg/kg intravenously every 12 h the first three days after HT then switched to valganciclovir 900 mg by mouth daily for CMV prophylaxis, a single strength sulfamethoxazole/trimethoprim by mouth daily for pneumocystis jirovecii prophylaxis and nystatin liquid 500,000 units by mouth after meals and before bedtime for oral candidiasis prophylaxis. Valganciclovir and sulfamethoxazole/trimethoprim were discontinued 6-months and one-year post-HT, respectively. For his routine immunosuppression regimen, he was started on methylprednisolone 125 mg intravenously every 8 h for 3 doses followed by oral prednisone taper to off at 6-months post-HT, mycophenolate mofetil with initial dose of 1000 mg orally every 12 h which was gradually tapered to 250 mg twice daily, and tacrolimus with the dose adjusted based on a goal serum drug level of 10–12 ng/ml the first six months, titrated down to 8–10 ng/ml for the subsequent six months, then 6–8 ng/ml thereafter (Fig. 1). No other treatment for rejection were administered (i.e., basiliximab or thymoglobulin). In addition, his post-HT medications included aspirin 81 mg daily, diltiazem controlled-delivery 360 mg po daily, lisinopril 5 mg po daily, pitavastatin 1 mg po three times weekly, bupropion 300 mg po daily, sertraline 50 mg po daily, famotidine 20 mg po daily as well as calcium carbonate 500 mg po twice daily, magnesium oxide 800 mg po daily and ferrous sulfate 325 mg po daily.

**Fig. 2:**
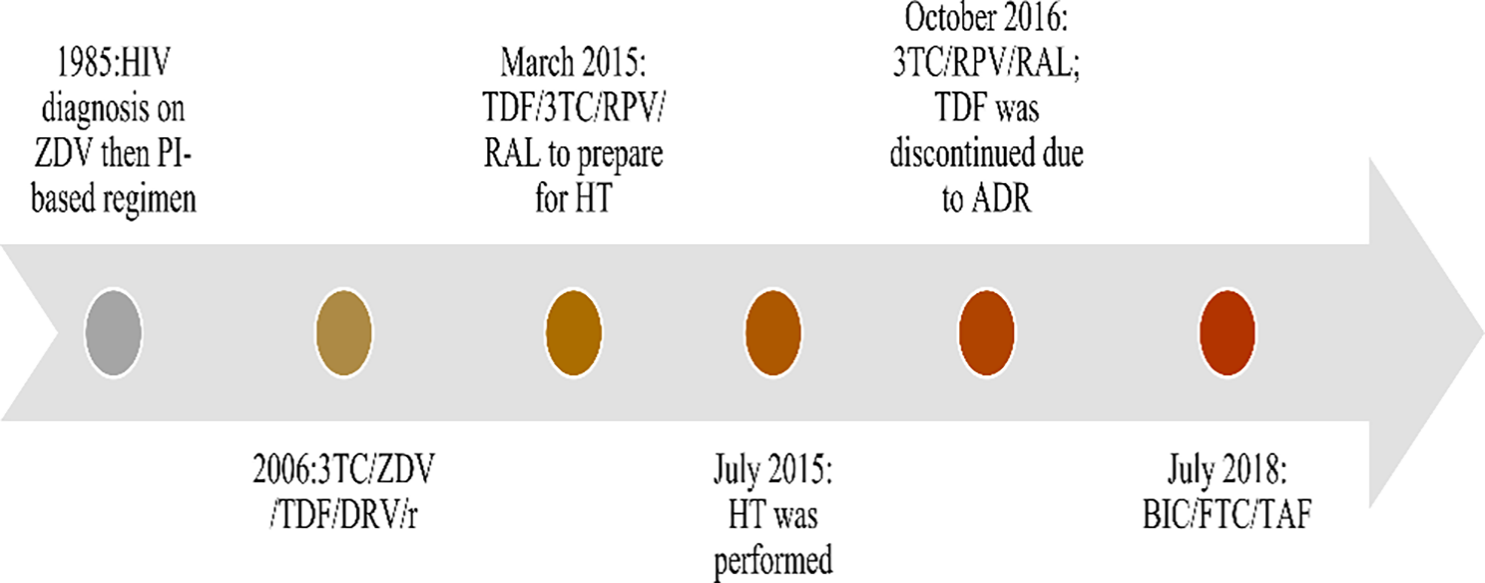
A graphical timeline with key events and antiretroviral changes **Abbreviations**: ADR, adverse drug reaction; BIC, bictegravir; DRV, darunavir; FTC, emtricitabine; HIV, human immunodeficiency virus; HT, heart transplantation; PI, protease inhibitors; RAL, raltegravir; r, ritonavir; TAF, tenofovir alafenamide; RAL, raltegravir; RPV, rilpivirine, TDF, tenofovir disoproxil fumarate; ZDV, zidovudine; 3TC, lamivudine

Seventy-six months from the HT, the patient remained in good health. There was marked improvement in cardiac function shortly after the transplantation, which has continued to date and he has not had any AIDS-defining illnesses or opportunistic infections nor major adverse effects from ART since the HT. As anticipated, post-HT, his absolute CD4 count declined to 297 cells/mm^3^ the day of the transplantation, but he exhibited preserved percent (33%) increased to 656 cells/mm^3^ (47%) four months later and has since remained above 500 cells/mm^3^. Serial HIV-1 RNA blood levels continue to be below the limit of quantification (< 40 copies of HIV-1 RNA per milliliter of plasma). CD4 count, CD4%, and quantitative HIV-1 RNA overtime are shown in Fig. 1. The patient has been maintained on lamivudine, rilpivirine and raltegravir and a low-dose standard immunosuppression regimen with tacrolimus and mycophenolate mofetil. Tenofovir disoproxil fumarate was discontinued 15-months post-HT due to concern of declining renal function. Three years post-HT, his ART was simplified to a single daily tablet containing bictegravir 50 mg, emtricitabine 200 mg, and tenofovir alafenamide 25 mg. Figure 2 presents a graphical timeline with key events and antiretroviral changes.

Few post-HT complications developed over the 76-month follow-up period. At 22-months post-HT, the patient was noticed to have an abnormal prostate exam and elevated prostate specific antigen of 8.1 ng/ml (from a baseline of 3.1 ng/ml in the previous year). Biopsy of the prostate demonstrated a Gleason 3 + 3 adenocarcinoma of the prostate and he underwent robotic-assisted laparoscopic prostatectomy. He also developed basal cell carcinoma for which mycophenolate was safely stopped 44-months post-HT with less new cancerous lesions thereafter. In addition, the patient experienced oral thrush unresponsive to nystatin and treated with fluconazole, multiple episodes of bilateral maxillary sinusitis treated with oral antibiotics and idiopathic neuropathy initially treated with topiramate and later managed with pregabalin.

The patient developed only one asymptomatic episode of acute cellular rejection (International Society for Heart and Lung Transplantation (ISHLT) grade 2R/3A) at 26-months post-HT, which was treated with prednisone 50 mg orally twice daily for six doses. While the initial two annual heart catheterizations revealed no evidence of cardiac allograft vasculopathy (CAV), beginning at 36-months post-HT, he developed asymptomatic 2-vessel CAV which was initially mild (most severe coronary stenosis 40% in both the left anterior descending and circumflex arteries). This CAV became more severe with diffuse disease at 48-months post-HT onward (most severe stenosis 90% in the small caliber left circumflex artery), but with no targets for revascularization and with no obvious exertional symptoms, in part conceivably related to chronic peripheral neuropathy which limits his physical activity. In response to his progressive CAV, his tacrolimus monotherapy was augmented with the addition of a mammalian target of rapamycin inhibitors (mTOR) (initially everolimus followed by sirolimus at 52- and 60-months post-HT, respectively), which was subsequently discontinued at 63-months post-HT given the patient’s peripheral feet neuropathy became a primary limiting symptom. Thus, prednisone 5 mg orally daily was added, with stable CAV documented by coronary angiography at 60- and 65-months post-HT. His current cardiac regimen includes tacrolimus, prednisone, vitamin C, vitamin D, evolocumab (neuropathy worsened on statin), losartan, diltiazem, and aspirin. No other major complications have developed as of November 2021.

## Discussion

This case describes the successful clinical outcome of an HIV-positive HT recipient after 76 months of follow-up. Patient is alive with a sustained normalization of cardiac function. Despite transitioning the ART regimen prior to HT listing, his serial HIV-1 RNA blood levels have been constantly below the limit of quantification, and his CD4 count has persistently remained above 500 cells/mm^3^ with the exception of the day of transplantation (Fig. 1). He has not had any AIDS-defining illnesses or opportunistic infections even under more intense immunosuppression that involved glucocorticoids, calcineurin inhibitor (CNI), and an antimetabolite during the first few months after HT as well as oral glucocorticoids during one acute rejection episode. While no CAV occurred early post-HT, immunosuppression was weaned due to prostate and skin cancers, but then readjusted in response to subsequent diffuse CAV development. HIV-positive status and ART did not appear to adversely affect the long-term graft function. No major adverse reactions to ART or immunosuppressants were observed aside from neuropathy with mTOR inhibitor therapy.

In contrast to liver and kidney transplantation, only a small number of case reports have been published for HT in HIV-positive individuals. Due to the paucity of clinical data, careful evaluation and selection of a HT candidate in the setting of HIV infection is warranted. Essential consideration should be given to pre-transplant HIV-RNA viral load and CD4 count, presence of opportunistic infections and potential for DDIs with following initiation of HT therapies. In our patient, preoperative evaluation demonstrated a suppressed viral load and no history of opportunistic infections. These factors are essential to predict a successful clinical outcome post transplantation. Table 1 summarizes the baseline characteristics and clinical outcomes of previously published reports for HIV-positive HT recipients.

Similar to previously reported cases, this case is not free from complications. During the follow-up period, there was one episode of ISHLT grade 2R/3A, which necessitated treatment with pulse oral corticosteroid therapy. This was the only episode of treated acute cellular rejection. The rejection was presumed to be due to subtherapeutic tacrolimus levels after empiric tacrolimus dose reduction in the setting of fluconazole initiation. It should be noted that rejection rates are higher in kidney and liver transplant recipients with HIV compared to recipients who do not have HIV [11]. Three years post-HT, mild CAV developed and progressed the following year. While the weaning of the immunosuppression due to his cancers may be a possible risk factor, there are concerns about possible increased risk of CAV in HIV-positive recipients. However, in a recent retrospective analysis, rates of CAV in HIV-positive recipients (32%) were similar to CAV rates in overall HT population (29.3%) up to 5 years of follow-up [12]. Due to previous statin and ezetimibe intolerance, dyslipidemia was initially treated with pitavastatin and later transitioned to evolocumab. Hypertension was managed with losartan and diltiazem. The patient’s post-HT course has also been complicated by adenocarcinoma of the prostate and cutaneous basal cell carcinoma, which were likely related to immunosuppression as well as hormone replacement with testosterone and family history of prostate cancer. Of note, HIV infection does not appear to increase the risk of *de novo* malignancy after kidney and liver transplantation relative to non-HIV infection status [13].

Four months prior to transplantation, the patient’s ART was changed from a protease inhibitor-based regimen including lamivudine, zidovudine, tenofovir disoproxil fumarate, and darunavir/ritonavir to tenofovir disoproxil fumarate, lamivudine, rilpivirine, and raltegravir for pre-transplant consideration. This is primarily due to the inhibition of CYP3A4-mediated tacrolimus metabolism by the protease inhibitor ritonavir [14]. For more information about drug-drug interactions between immunosuppressive agents and antiretroviral in HIV-infected patients after solid organ transplantation, reader can refer to this review article [15]. No pharmacokinetic interactions were found between his new ART and his immunosuppressants; however, other significant interactions exist for commonly used ART. Rilpivirine requires an acidic environment for optimal absorption; therefore, acid suppressing medications can significantly reduce rilpivirine absorption and exposure. Our patient was taking famotidine which can be concomitantly taken; however, it should be dosed 12 h before or 4 h after rilpivirine. Moreover, polyvalent cations such as calcium, magnesium, aluminum, and iron can significantly decrease raltegravir absorption and exposure. Our patient was taking calcium carbonate, ferrous sulfate and magnesium oxide which can be concomitantly used if raltegravir is given 2 h before or 6 h after these products. Administration times of polyvalent cations and famotidine were adjusted during his admission and he was educated to follow these instructions at discharge. Diltiazem can increase tacrolimus trough concentrations due to inhibition of cytochrome P-450 CYP3A-mediated tacrolimus metabolism. Tacrolimus trough concentrations were carefully monitored with subsequent dose optimization during his follow-up visits.

Three years post-HT, his ART was switched to a single daily tablet containing bictegravir, emtricitabine and tenofovir alafenamide. This regimen has the advantage of reduced pill burden, does not have a food requirement, and does not interact with acid suppressants. Although bictegravir is a CYP3A and UGT1A1 substrate and tenofovir alafenamide is a P-gp substrate, there was no clinically important drug interaction with his other medications. This same interaction previously mentioned with polyvalent cations also exists with bictegravir and warranted consideration. Although tenofovir disoproxil fumarate was previously discontinued due to renal dysfunction, his renal function has improved and allows the use of the newer tenofovir prodrug, tenofovir alafenamide, which is less likely to have nephrotoxicity than the older prodrug.

The rationale against HT in people with HIV arises from speculation that the use of immunosuppressants may increase the risk of AIDS-defining illness, the risk of drug interactions between ART and immunosuppression, and a paucity of data on survival and clinical outcomes. Consistent with previously reported cases (Table 1), our patient has not had AIDS-defining illnesses or opportunistic infections. In regards to DDIs, protease inhibitors, non-nucleoside reverse transcriptase inhibitors and cobicistat-boosted regimens have the most significant and clinically relevant drug interactions with immunosuppression medications [11]. In contrast, nucleoside reverse transcriptase inhibitors, non-boosted integrase inhibitors and rilpivirine are all devoid of these pharmacokinetic interactions [11]. Our patient is now on an antiretroviral regimen free of protease inhibitors, pharmacokinetic boosters (ritonavir, cobicistat), and non-nucleoside reverse transcriptase inhibitors. The current regimen has low potential to interact with his immunosuppression regimen. Lastly, a recent large retrospective analysis was conducted in the US using nationwide Organ Procurement and Transplantation Network data between 2014 and 2017, 41 HIV-positive HT recipients were identified [12]. When matched to 41 HIV negative HT recipients for idiopathic dilated-cardiomyopathy, there was no significant difference in post‐HT survival for up to 5 years. Moreover, rates of CAV and malignancy in HIV-positive HT recipients were comparable to the overall HT population. This study demonstrated that more than 80% of HT centers in the US had not performed a HT in an HIV-positive patient. Similarly, in a more recent retrospective analysis of the thoracic United Network for Organ Sharing database, 75 HIV-positive HT recipients and 29,848 HIV-negative HT recipients were identified. The 1-year and 5-year survival rates were comparable with no significant difference [16].

In summary, our case supports that HIV-1 positive status does not preclude HT in carefully selected individuals. In this case, the clinical outcome was comparable to the general population with HIV as well as those receiving HT. First, no opportunistic infections developed in the setting of HT immunosuppressive therapy. While concerns have been raised about the effects of immunosuppression in people with HIV, this patient’s virologic suppression through the peritransplant period mitigated risk of opportunistic infection. In addition, his underlying HIV and concomitant ART did not appear to negatively impact early or intermediate-term graft function. Furthermore, his immunosuppressive transplant regimen did not affect his HIV-related outcomes despite modification of his ART prior to transplant. Even though it is less frequently performed and reported than liver and kidney transplantation, HT in this population appears to be successful in carefully chosen and well-managed patients with virologic suppression in whom DDIs have been mitigated.

## Data Availability

Data sharing is not applicable to this article as no datasets were generated or analysed during the current study.

## List of abbreviations

ART: Antiretroviral therapy
CAV: Cardiac allograft vasculopathy
CMV: Cytomegalovirus
CVD: Cardiovascular disease
DDIs: Drug-drug interactions
HF: Heart failure
HIV: Human immunodeficiency virus
HT: Heart transplant
